# Development of a Dual-Layer Structure for Cymbal Transducer Arrays to Achieve a Wider Bandwidth

**DOI:** 10.3390/s22176614

**Published:** 2022-09-01

**Authors:** Jahnavi Mudiyala, Hayeong Shim, Donghyun Kim, Yongrae Roh

**Affiliations:** School of Mechanical Engineering, Kyungpook National University, Daegu 41566, Korea

**Keywords:** cymbal transducer, arrays, wideband, dual-layer structure, mutual radiation impedance

## Abstract

Cymbal transducers are typically grouped and arranged in planar arrays. For projector arrays, a wide bandwidth on the transmitting voltage response (TVR) spectrum is required for better underwater communication and data transmission within a short time. The purpose of this study is to develop a wideband cymbal array by controlling the center-to-center (CTC) spacing between the cymbal transducers in the array. In the practical design of the array, due to the arrangement of elements in one layer, the minimum CTC spacing between the cymbals is constrained to the diameter of the cymbals in use. To overcome this limitation, we propose a new dual-layer array structure. Finite element analysis of the cymbal array showed that the bandwidth was generally inversely proportional to the CTC spacing. We explained the mechanism of this relationship using a theoretical analysis of the mutual radiation impedance between the cymbals in the array. Subsequently, we identified the optimum CTC spacing to achieve the widest possible bandwidth for the cymbal array. The validity of the wideband array design was verified through the fabrication and characterization of prototype arrays. We confirmed that the two-layered arrangement could significantly widen the bandwidth of the cymbal array while maintaining the TVR above a specified level.

## 1. Introduction

Underwater wireless sensor network systems are essential for modern underwater communication and detection [1]. Broadband arrays are in high demand for under-water communication applications owing to their high-speed data transmission and detection capabilities [2,3,4]. This study presents the development and fabrication of a cymbal array focusing on broadband acoustic characteristics.

A cymbal transducer is a miniaturized version of class-V flextensional transducers. The transducer comprises a piezoceramic disc sandwiched between two metal caps with a cavity inside, wherein the cap functions as a mechanical transformer [5]. The operating principle of this transducer can be explained by the electroacoustic transduction mechanism; the small radial extensional motion of the ceramic disk is transmitted to the large flexural motion of the cap [6,7]. Owing to the low efficiency and high-quality factor of these transducers [8], they are typically used as arrays [9,10,11]. Cymbal arrays can amplify the transmission power and widen the frequency bandwidth [12,13,14].

Numerous studies have been conducted to identify methods for widening the bandwidth of cymbal arrays. Tressler et al. [6] first fabricated arrays using nine 16.1 kHz cymbal elements via two different mounting methods, and then analyzed their frequency responses. Newnham et al. [15] constructed a 5 × 20 array prototype, calculated the broadband response of the array, and compared its acoustic characteristics with those of a 3 × 3 array. Kim et al. [16] designed a 3 × 3 array with cymbal transducers which had three different frequencies to achieve a broadband frequency response. Previous studies have reported that several parameters affect the frequency response of cymbal arrays, such as the structural and material properties of individual cymbal transducers, array mounting conditions, number of array elements, polarity of the elements, and center-to-center (CTC) spacing between the elements in the array [6,8,15,17]. The effects of most of these parameters have been investigated previously. However, the effect of CTC spacing has not been analyzed comprehensively, although it is one of the most effective parameters for controlling the frequency response [18,19,20].

Hence, in this study, we investigated the effect of CTC spacing on the broadband characteristics of the array. Zhang et al. [18] analyzed this interaction in a cymbal array and concluded that the flat transmitting voltage response (TVR) spectrum of the array was due to the narrow element spacing. They examined the effect of CTC spacing on the TVR spectrum using the finite element method (FEM) as well [19]. Recently, Kim et al. [20] analyzed the frequency response of a cymbal array and optimized the CTC spacing and center frequency of the constituent cymbals in order to maximize the bandwidth. They discovered that reducing the CTC spacing yielded a flatter response in the TVR spectrum irrespective of the center frequency of the cymbal transducers. Although these studies investigated the effect of CTC spacing on the TVR spectrum, the analyses were based on trial-and-error using the FEM, and a clear explanation was not provided for this phenomenon. In addition, the cymbal transducers were arranged in a planar array; however, the arrangement of cymbals on one layer constrained the minimum CTC spacing between the array elements to the diameter of the cymbals.

In this study, to overcome the limitation of minimum CTC spacing in an array, a new dual-layer array arrangement was proposed to accommodate a more flexible assignment of CTC spacing between the array elements. Unlike existing single layer arrays, where the spacing between the elements is restricted, the dual-layer structure allows for the flexible adjustment of the spatial layout of the array. The operation mechanism of the dual-layer structure was explained in detail, and the arrangement of cymbal transducers in this structure was used to attain a wider bandwidth on the TVR spectrum. This kind of arrangement for improving the bandwidth of the cymbal array has not been studied before.

The acoustic characteristics of a 3 × 3 cymbal array arranged in two layers with various CTC spacings, as an example of the dual-layer structure, were analyzed using the FEM. The effect of CTC spacing on the TVR spectrum was explained by the mutual radiation impedance between the array elements. Subsequently, the CTC spacing was optimized to maximize the bandwidth of the array. The advantage of the new arrangement over the conventional one-layered array was presented with the results. To validate the results, prototypes of 3 × 3 and 5 × 5 cymbal arrays with the dual-layer structure were fabricated. The acoustic characteristics of the prototypes were measured and compared.

In the following section, the dual-layer structure is introduced, and the effect of the dual-layer arrangement parameters, such as the vertical and CTC spacings between array elements on the TVR spectrum of the array, is analysed using the FEM. Following this, the mechanism of the effect of CTC spacing is explained by analysing the interaction between the elements. Then, the optimum CTC spacing is obtained to achieve the widest possible bandwidth of the array. Finally, the effectiveness of the dual-layer structure is verified by comparing actual measured values with the results of the finite element analysis (FEA).

## 2. The Dual-Layer Arrangement of the Cymbal Array

**Figure 1:**
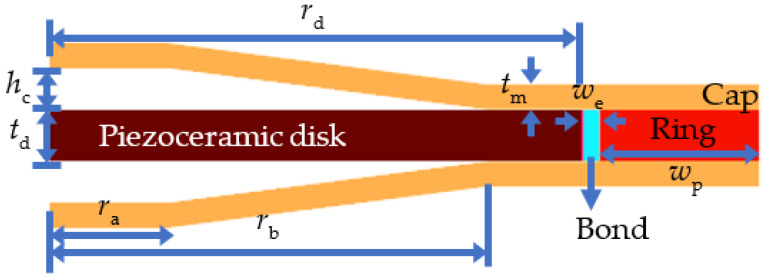
Finite element model of the cymbal transducer.

Figure 1 shows a two-dimensional axisymmetric view of the cymbal transducer used in this study. The cymbal transducer comprises a PZT-5A ceramic disk and brass caps. The PZT disk was sandwiched between two brass caps, which were bonded to each other. The PZT disk vibrates in a radial motion, and the brass caps connected to the disk move in a vertical direction radiating sound pressure waves to the surroundings. A plastic ring was attached to the disk via a 0.3-mm-thick bond layer to improve the structural robustness of the transducer. The cymbal transducers were coated with a 0.3-mm-thick rubber layer (RTV-3460 (Elkem, Oslo, Norway)) for insulation.

The center frequency of this transducer was 12.7 kHz (*f*_1_). The structural parameters of the transducer shown in Table 1 were optimized to achieve the widest bandwidth using the method described in [8]. The acoustic performance of the cymbal transducer was analyzed by the FEM while varying the structural parameters in Table 1. Results of the FEA were analyzed to determine the optimal values of the parameters with the objective of maximizing the bandwidth while constraining the peak TVR level. Table 1 lists the optimized dimensions of the structural parameters. The material properties of the of PZT-5A, brass caps and coating layer are same as those presented in [21]. The density, Young’s modulus, and the Poisson’s ratio of the ring are 1300 kg/m^3^, 4 GPa, and 0.33, respectively.

**Figure 2:**
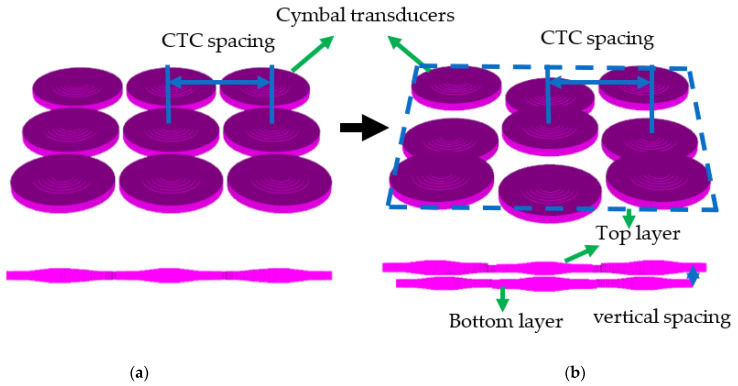
Arrangement of the 3 × 3 cymbal arrays in: (**a**) one layer; (**b**) two layers.

The objective of this study is to analyze the effect of CTC spacing on the frequency response of the cymbal array, and to design a wideband array based on this analysis. In conventional cymbal arrays, the array elements are arranged on a single plane, as shown in Figure 2a. Because the cymbal elements are arranged in one layer, the minimum CTC spacing is confined to the diameter of the individual cymbal transducer. To address this issue, we propose a new arrangement of cymbal transducers in a dual-layer structure, as shown in Figure 2b, to allow for a more flexible assignment of CTC spacing between the elements.

To verify the efficacy of the two-layered structure, we analyzed a 3 × 3 array of cymbal elements. Figure 2 shows the finite element models of the one-layered and two-layered 3 × 3 arrays. The two-layered 3 × 3 cymbal array comprised five and four elements placed on the top and bottom layers, respectively. These two layers were separated by a certain amount of vertical spacing. The CTC spacing in the two-layered array refers to the horizontal spacing between the centers of the elements. The horizontal CTC spacing is measured as the horizontal distance between the centers of the neighboring cymbals.

**Figure 3:**
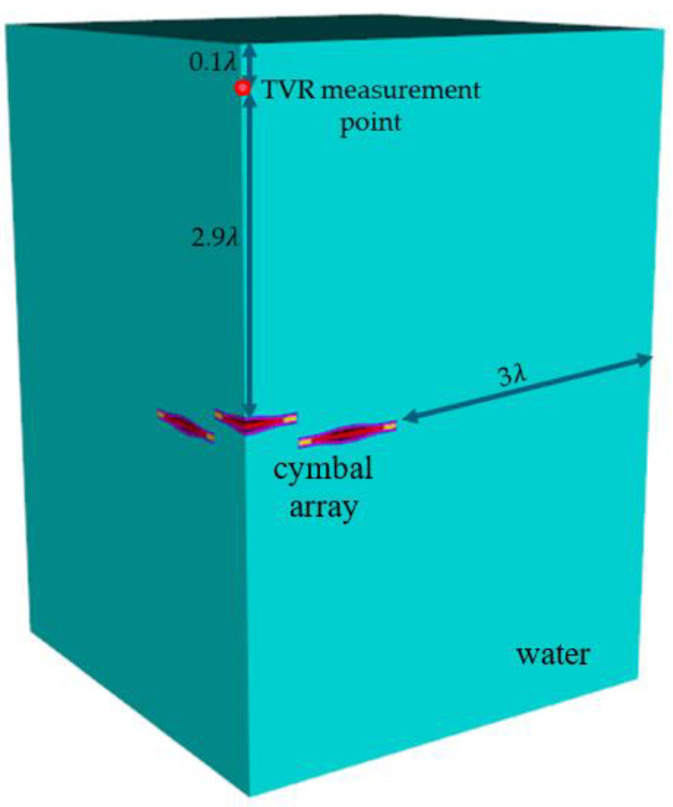
Finite element model of the immersed 3 × 3 cymbal array.

The acoustic characteristics of the array models were analyzed using the commercial FEA software PZFlex^®^ [22]. Owing to the symmetrical structure, only a quarter of the three-dimensional model of each array was analyzed to conserve time and memory. All cymbal elements in the array were identical, with the same center frequency *f*_1_; these dimensions are presented in Table 1. The element size in the finite-element model was 0.15 mm. To analyze their underwater performance, the array was surrounded with water of 3*λ* length on all sides, as shown in Figure 3. Here *λ* is the wavelength of the acoustic wave in water at *f*_1_. The boundaries of water were enforced with acoustic absorption conditions. The TVR spectrum of the array was analyzed at a far-field point from the array, which was 2.9*λ* from the apex of the center element in the array. The frequency scale of all the acoustic characteristics was normalized to *f*_1_.

### 2.1. The Effect of Vertical Spacing between Layers of the Two-Layered Structure

**Figure 4:**
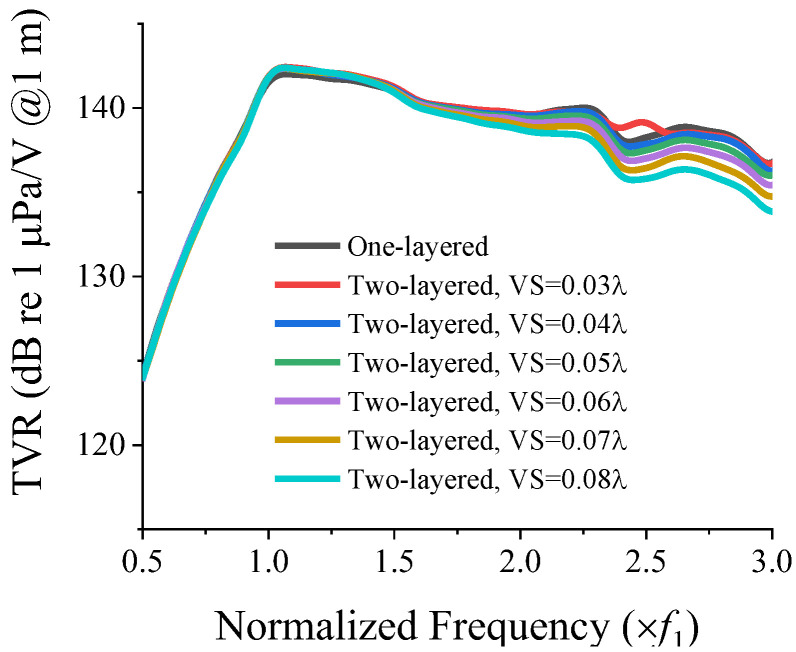
TVR spectra of the 3 × 3 cymbal array arranged in one-layered and two-layered structures with CTC spacing of 0.23*λ*, and vertical spacing (VS) from 0.03*λ* to 0.08*λ*.

To analyze the effect of the two-layered structure on the frequency response of the cymbal array, we first investigated the effect of the vertical spacing between the layers on the acoustic characteristics of the array. All the dimensions are presented as multiples of *λ*. With the CTC spacing of 0.23*λ*, which corresponded to the closest spacing for the one-layered array, the TVR spectrum of the two-layered 3 × 3 array was analyzed for various vertical spacings, and the results are presented in Figure 4. The vertical spacing was varied from 0.03*λ* to 0.08*λ* at an interval of 0.01*λ*, where 0.03*λ* was the minimum vertical spacing that could be assigned to avoid the overlapping of the cymbal elements, while the CTC spacing was varied in the two-layered structure. Subsequently, quantitative acoustic characteristics were extracted from the TVR spectra, and the results are presented in Table 2. The fractional bandwidth (FB) is the ratio of the −3 dB bandwidth to the center frequency.

The one-layered array with the closest CTC spacing was regarded as the basic model. As shown by the results in Figure 4 and Table 2, the change in the FB from the basic model was less than 5% for the vertical spacing that was less than 0.07*λ*. Considering analysis and manufacturing errors, it is reasonable to conclude that for a vertical spacing of 0.06*λ* or less, the two-layered arrangement of cymbal transducers yielded an almost identical TVR spectrum to that of the basic model. For ease of manufacturing, we selected a vertical spacing of 0.04*λ* for the 3 × 3 dual-layer array arrangement.

### 2.2. The Effect of CTC Spacing on the Acoustic Characteristics of the Two-Layered Array

**Figure 5:**
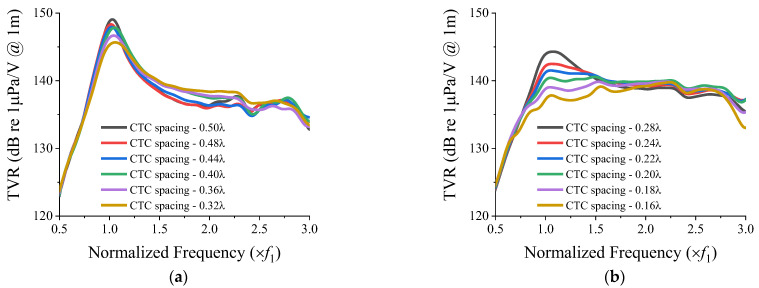
Effect of CTC spacing on the TVR spectrum of the 3 × 3 two-layered array: (**a**) for CTC spacing from 0.50*λ* to 0.32*λ*; (**b**) for CTC spacing from 0.28*λ* to 0.16*λ*.

With the vertical spacing fixed, we analyzed the effect of the CTC spacing on the TVR spectrum of the 3 × 3 two-layered array by varying the spacing from 0.5*λ* to 0.16*λ* using the FEM. The minimum CTC spacing was set to 0.16*λ* to avoid the base of the metal cap of a cymbal element from overlapping with the ceramic of another cymbal element in the array. The TVR spectra for different CTC spacings are presented in Figure 5.

As the CTC spacing decreased, the peak TVR level decreased. The FB and other quantitative acoustic characteristics were extracted from the TVR spectra shown in Figure 5, and the results are presented in Table 3.

As shown in Table 3, as the CTC spacing decreased and the peak TVR level decreased, whereas the FB increased. The FB reached its maximum at a certain point, and decreased after that point. According to the results, the CTC spacing of 0.20*λ* afforded the widest bandwidth for the cymbal array. The reasoning for this mechanism is explained in the next section.

The maximum FB was observed for the CTC spacing of less than 0.23*λ*, which corresponds to the diameter of the cymbal transducer, and the minimum CTC spacing achievable when the cymbal elements are arranged in one layer. However, the broadening of the bandwidth was hampered by the size of the transducer and the arrangement of the array elements in one layer, which limited the further reduction in the CTC spacing. The dual-layer arrangement eliminated this limitation and allowed the bandwidth to be widened further, thus demonstrating the efficacy of the dual-layer structure.

## 3. The Mutual Radiation Impedance with Varying CTC Spacing

The effect of CTC spacing on the acoustic characteristics of the two-layered cymbal array (Figure 5 and Table 3) can be explained by the acoustic loading on the transducer elements. This acoustic loading includes the self-radiation impedance of the array elements, which reflects the effect of the surroundings, and the mutual radiation impedance between the elements. Compared with other transducers, cymbal transducers differ significantly in their resonant frequencies in air and in water [23]. Due to the cap’s compliant nature, acoustic loading significantly affects the operation of cymbal transducers. The effect of CTC spacing on the TVR spectrum can be explained by the mutual radiation impedance between the elements in the array. 

**Figure 6:**
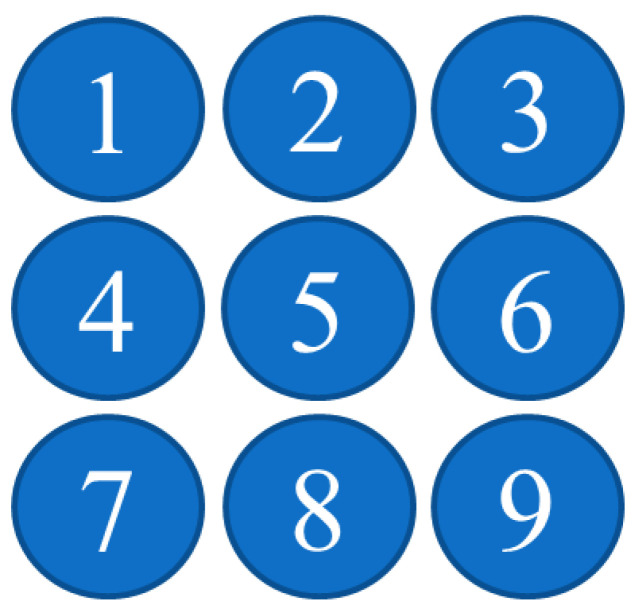
Top view of the 3 × 3 cymbal array.

Figure 6 is a simplified view of a 3 × 3 cymbal array. The radiation impedances were analyzed considering cymbal elements as flat circular piston sources [24]. Because the spherical section of the cap contributes significantly to the radiation from the cymbal transducer, the radius of the circular piston was regarded as the base radius ($r_{b}$) of the metal cap [21].

The self- and mutual radiation impedances of the circular piston sources as functions of frequency were calculated using Equations (1) and (2). Equation (1) expresses the self-radiation impedance of the *m*^th^ element $\left(Z_{m}^{s}\right)$ [25], and Equation (2) expresses the mutual radiation impedance between the *m*^th^ and *n*^th^ elements ($Z_{mn}^{t}$) in the array [1,6].

(1) $$Z_{m}^{s}=R_{m}^{s}+jX_{m}^{s}=\rho cA\left(1-\frac{2J_{1}\left(2kr_{b}\right)}{2kr_{b}}+j\frac{2H_{1}\left(2kr_{b}\right)}{2kr_{b}}\right)$$

(2) $$Z_{mn}^{t}=R_{mn}^{t}+jX_{mn}^{t}=\frac{\rho ck^{2}A^{2}}{2\pi }\left(\frac{sin\left(kd_{mn}\right)}{kd_{mn}}+j\frac{cos\left(kd_{mn}\right)}{kd_{mn}}\right)$$

where

- k = wave number,
- $A=\pi r_{b}^{2}$,
- $d_{mn}$ = CTC spacing between the *m*^th^ and *n*^th^ elements,
- ρ = density of water,
- c = sound velocity in water,
- $J_{1}$ = first-order Bessel function of the first kind,
- $H_{1}$ = first-order Struve function.

**Figure 7:**
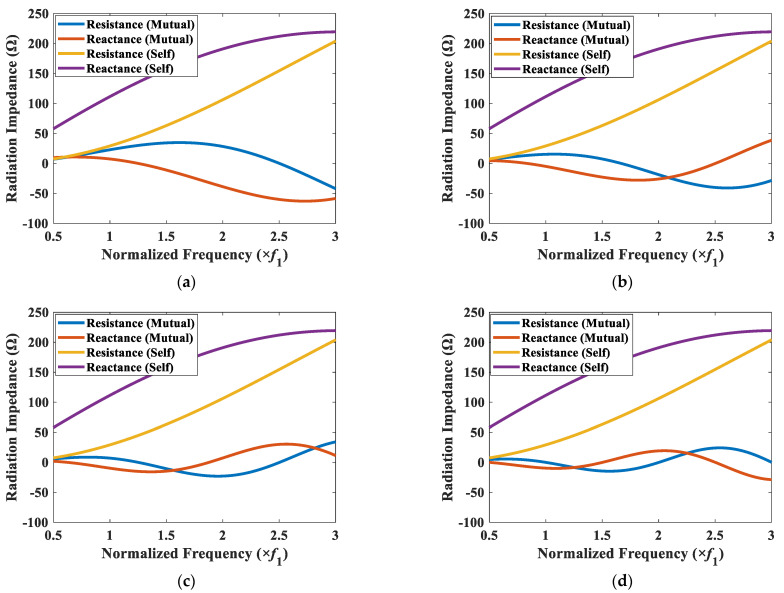
Self-radiation impedance of cymbal 1, and mutual radiation impedance between cymbals 1 and 2 for different CTC spacings: (**a**) 0.2*λ*; (**b**) 0.3*λ*; (**c**) 0.4*λ*; (**d**) 0.5*λ*.

As a simple case to check the radiation impedances, the variation of $Z_{1}^{s}$ and $Z_{12}^{t}$ as a function of frequency for the CTC spacings of 0.2*λ* to 0.5*λ* with an interval of 0.1*λ*, are presented in Figure 6, where the subscripts 1 and 2 denote cymbal 1 and cymbal 2 from Figure 5, respectively. The self-radiation impedance of the transducer was independent of the CTC spacing, whereas the mutual radiation impedance varied significantly with the change in the CTC spacing. In Figure 7a for the CTC spacing of 0.2*λ*, the mutual radiation resistance and reactance varied from −41.5 W to 35.0 W and from −58.6 W and 7.8 W, respectively. In contrast, in Figure 7d for the CTC spacing of 0.5*λ*, they varied from −14.7 W to 24.2 W and from −28.7 W and 19.4 W, respectively. For a CTC spacing of 0.5*λ*, the magnitude of the mutual radiation impedance was much smaller than that of the self-radiation impedance, which implied a weak interaction between the cymbals in the array. When the CTC spacing was 0.2*λ*, the variation of the magnitude of the mutual radiation impedance was larger than that when the CTC spacing was 0.5*λ*, which implied a stronger interaction between the cymbals occurred as the CTC spacing decreased.

**Figure 8:**
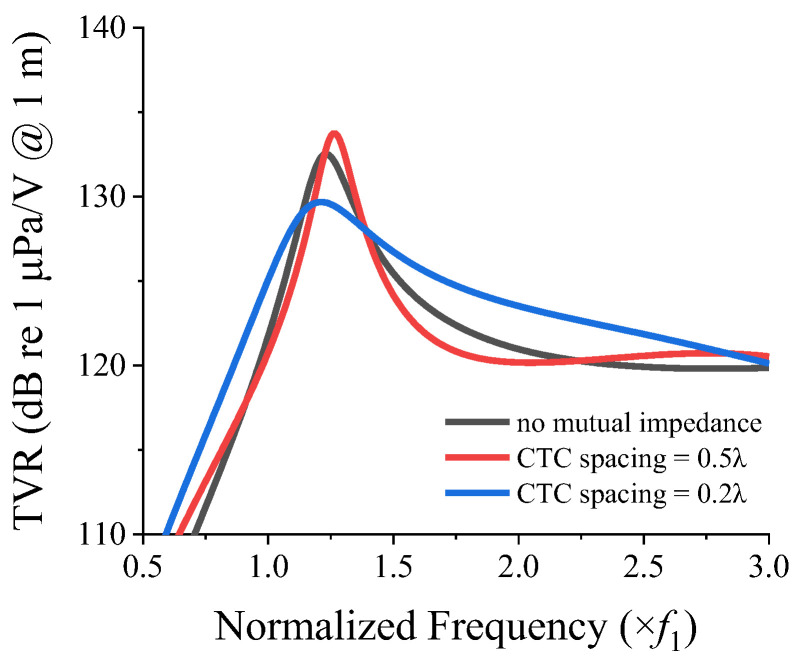
TVR spectra of a cymbal transducer with mutual radiation impedance added to the radiation load for different CTC spacings from its adjacent cymbal transducer.

To understand the effect of the mutual radiation impedance on the transmitting characteristics of the cymbal transducer array, we analyzed the TVR spectrum of cymbal 1 by using the equivalent circuit developed in [21]. All of the circuit parameters, except the radiation load, were the same as those presented in [21]. The radiation load of cymbal 1 was calculated as the summation of its self-radiation impedance $Z_{1}^{s}$, and the mutual radiation impedance $Z_{12}^{t}$ with cymbal 2. Figure 8 presents the TVR spectra of the cymbal 1 transducer when the CTC spacing from its neighboring cymbal was 0.5*λ* and 0.2*λ*. For comparison, Figure 8 also shows the TVR spectrum when no mutual radiation impedance was included in the radiation load.

When the cymbal transducers were closer to each other, the mutual radiation impedance between them reduced the peak TVR level. This implied that the mutual radiation impedance imposed a dampening effect on the TVR spectrum of the array, which led to a broader bandwidth. The TVR spectrum of a cymbal with a larger CTC spacing was similar to that of a cymbal that did not have the mutual radiation impedance.

**Figure 9:**
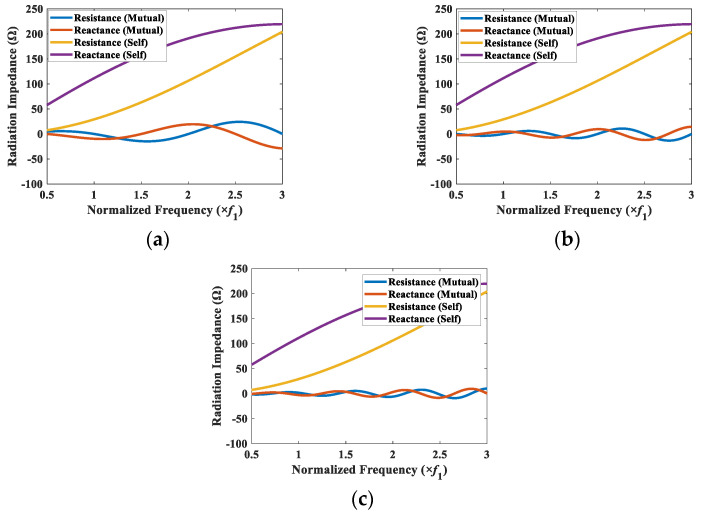
Self- and mutual radiation impedance of element 1 with its neighboring elements (CTC spacing of 0.5*λ*): (**a**) elements 1 and 2; (**b**) elements 1 and 7; (**c**) elements 1 and 9.

**Figure 10:**
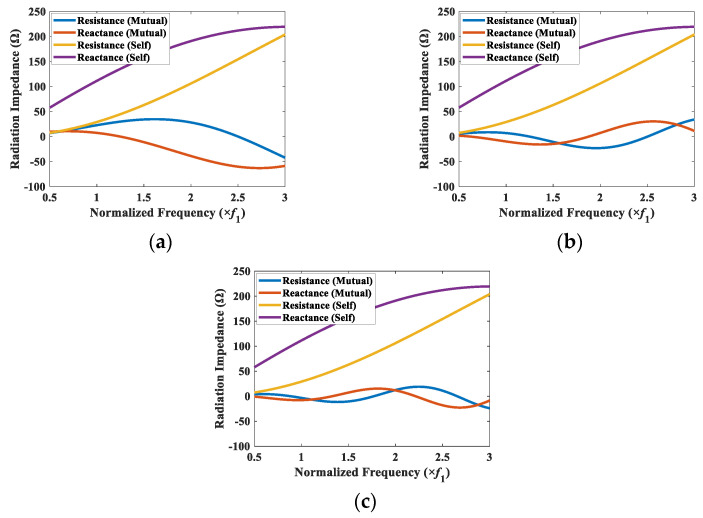
Self- and mutual radiation impedance of element 1 with its neighboring elements (CTC spacing of 0.2*λ*): (**a**) elements 1 and 2; (**b**) elements 1 and 7; (**c**) elements 1 and 9.

To elucidate the effect of the CTC spacing on the TVR spectrum of the array in detail, the mutual radiation impedance between a corner element (cymbal 1) and the other elements in the array was analyzed. The corner element was selected for the analysis owing to its varied spacing with the other elements in the array. The mutual radiation impedances between elements 1 and 2, 1 and 9, and 1 and 7 were calculated for the CTC spacings of 0.5*λ* and 0.2*λ*, and the results are presented in Figure 9 and Figure 10. In Figure 9a, the range of mutual radiation resistance was from −14.7 Ω to 24.2 Ω, and that of mutual radiation reactance was from −28.7 Ω to 19.4 Ω. In Figure 9c, they were from −9.0 Ω to 10.0 Ω for the mutual radiation resistance and from −8.4 Ω to 10.0 Ω for the mutual radiation reactance. The results show that the magnitude of mutual radiation impedance due to the farther element in the array was smaller than the magnitude of mutual radiation impedance due to the closer element. This indicated that the interaction with the farther element was less impactful compared with that of the closer element.

The mutual radiation impedances of the corner element with all of the other elements in the array were superimposed based on Equation (3) to observe the complete pattern.

(3) $$Z_{m}^{t}=\overset{9}{\underset{n=1,n\neq m}{\sum }}Z_{mn}^{t}$$

where $Z_{m}^{t}$ is the total mutual radiation impedance on the $m^{\mathrm{th}}$ element due to all the other elements in the array.

**Figure 11:**
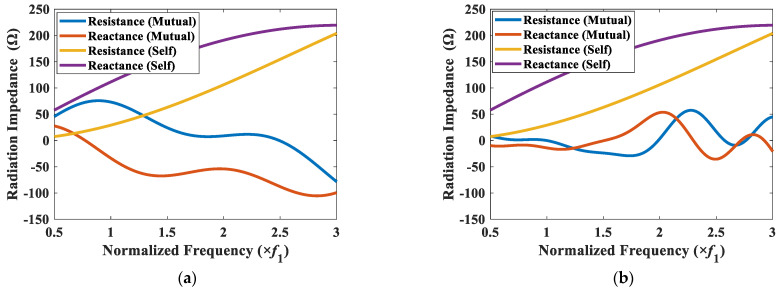
Self-radiation impedance $Z_{1}^{s}$ and total mutual radiation impedance $Z_{1}^{t}$ of the cymbal 1 transducer for CTC spacings of (**a**) 0.2*λ*; (**b**) 0.5*λ*.

The superposition of mutual radiation impedances on element 1 ($Z_{1}^{t}$) was calculated for CTC spacings of 0.2*λ* and 0.5*λ*, and the results are presented along with the self-radiation impedance $Z_{1}^{s}$ in Figure 11. The magnitude of the mutual radiation impedance is bigger than those in Figure 9 and Figure 10, but the overall pattern of the spectrum is similar. The mutual radiation impedance felt by the cymbal 1 was bigger when the CTC spacing from its neighboring cymbals was smaller, which indicated more interaction between the cymbals in the array.

**Figure 12:**
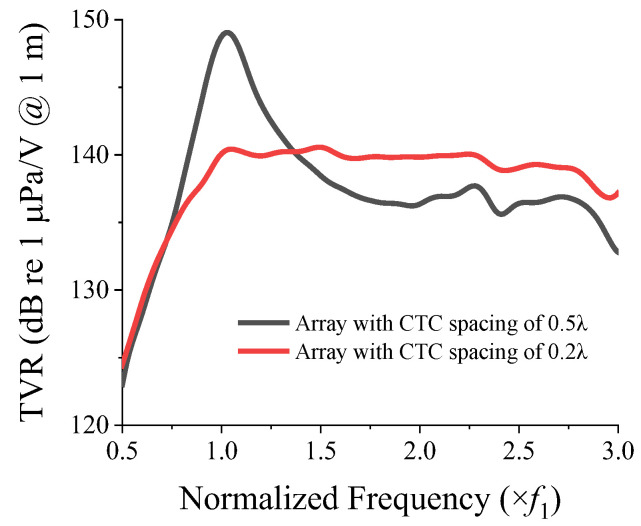
TVR spectrum of the two-layered array with CTC spacings of 0.5*λ* and 0.2*λ*.

When the interaction between the transducers in an array is negligible, the TVR spectrum of the array is expected to be similar to that of a single transducer with an enhanced source level. When the interaction is significant, the TVR spectrum will change accordingly. To highlight the effect of the interaction, a comparison of the TVR spectra of the 3 × 3 two-layered array for CTC spacings of 0.2*λ* and 0.5*λ* is shown in Figure 12. As shown in Figure 11a, the peak of the mutual radiation resistance occurred at *f*_1_, at which point the mutual radiation resistance was higher than the self-radiation resistance. This increase in the mutual radiation resistance caused the dampening of the TVR level at *f*_1_ for the CTC spacing of 0.2*λ* (see Figure 12). Figure 11b shows that the magnitude of the mutual radiation resistance at lower frequencies was approximately zero, thereby implying that for the CTC spacing of 0.5*λ*, the peak TVR level was not dampened. However, at higher frequencies, the mutual radiation resistance for 0.5*λ* was higher, whereas that for 0.2*λ* was almost equal to or less than zero, which implied no dampening in this frequency range, and hence, a flat curve.

However, by further reducing the CTC spacing, the TVR level at *f*_1_ dampened significantly to less than 3 dB from the peak level (Figure 5b). Thus, the flat spectrum was lost, which reduced the FB (Table 3). The abovementioned findings indicate that the FB increased as the CTC spacing decreased to a certain level, whereas the FB decreased thereafter. 

## 4. The Optimization of CTC Spacing between Array Elements to Maximize FB

**Figure 13:**
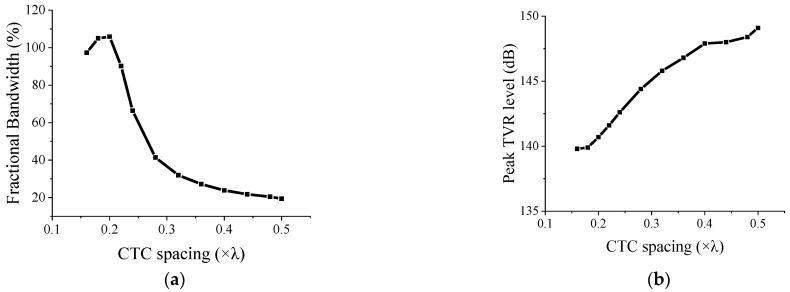
Acoustic characteristics of the 3 × 3 two-layered cymbal array vs. CTC spacing: (**a**) FB; (**b**) peak TVR level.

The variation in the peak TVR level and FB in the TVR spectrum were analyzed with respect to the CTC spacing based on the results presented in Table 3, and these results are presented in Figure 13.

The FB of the basic one-layered model was 79.2%. As our aim was to widen the bandwidth, the variation in CTC spacing was reduced to a range between 0.16*λ* and 0.24*λ*. The FB and peak TVR level were formulated as functions of the CTC spacing through a nonlinear regression analysis of the data within the abovementioned range. For improved accuracy, we employed fourth-order polynomials for the formulation instead of generally-used second- or third-degree polynomials. Equations (4) and (5) express the derived FB and peak TVR level functions, respectively. The CTC spacing is denoted by *x*.

(4) $$FB\left(x\right)=4763020x^{4}-3824690x^{3}+1129040x^{2}-145436.125x+7008$$

(5) $$\mathrm{peak}\;TVR\;level\left(x\right)=156250x^{4}-131250x^{3}+41312.5x^{2}-5730x+434.2$$

**Figure 14:**
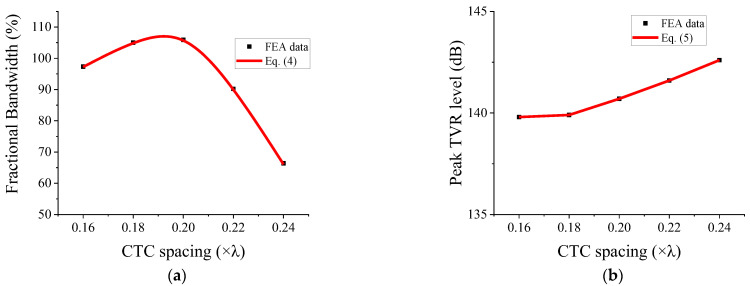
FEA data fitted with the fourth-order polynomial curve: (**a**) fractional bandwidth; (**b**) peak TVR level.

The derived functions are presented on the CTC spacing scale in Figure 14, along with the FB and peak TVR level data from Table 3.

Using the formulated functions for the FB and peak TVR level, the optimal value of the CTC spacing was determined to maximize the FB while constraining the peak TVR level. Typically, transducers for underwater applications need to have a peak TVR level higher than 140 dB [26,27]. Therefore, we set the peak TVR level constraint to be 0.5% higher than 140 dB to accommodate possible analysis errors. 0.5% was an arbitrary tolerance value we set for this study. Equation (6) shows the objective function of the optimization and the accompanying constraint.

Objective function: maximize the fractional bandwidth Constraint: peak TVR level ≥ 140.7 dB(6)

**Figure 15:**
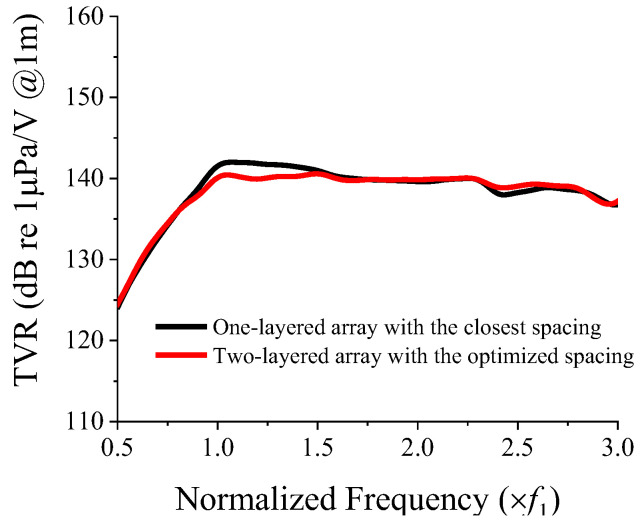
Comparison of the TVR spectrum of the one-layered 3 × 3 array with the closest spacing and that of the two-layered array with the optimized CTC spacing.

With the constraint on the peak TVR level set to be higher than 140.7 dB, the range of CTC spacing was limited to 0.2*λ*–0.24*λ*. In the CTC spacing range from 0.2*λ* to 0.24*λ*, Equation (5) is a monotonically decreasing function. Therefore, the maximum FB in this range was obtained when the CTC spacing was 0.2*λ*, and the corresponding peak TVR level was determined to be 140.7 dB. The optimum CTC spacing satisfying the aforementioned condition was therefore 0.2*λ*. The optimization process and derivation of equations presented in this section were conducted using MATLAB^®^. The TVR spectrum of the optimized model was compared with that of the basic model, as shown in Figure 15. The quantitative acoustic characteristics of both models are presented in Table 4. Figure 15 shows the efficacy of the two-layered arrangement for flattening the TVR spectrum of the array. By satisfying the TVR level constraint of 140.7 dB, the FB of the two-layered cymbal array with the optimized CTC spacing increased by 34%, compared with that of the one-layered array with the smallest spacing.

## 5. Validation of the Analysis

**Figure 16:**
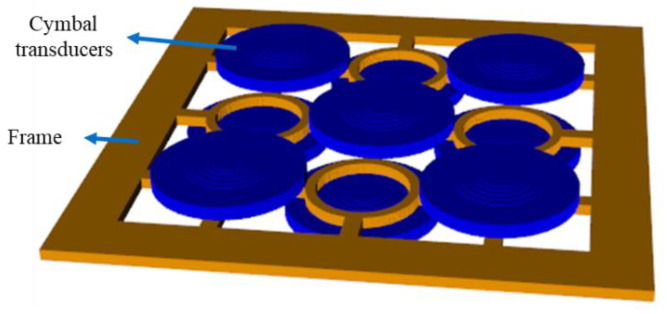
Finite element model of the two-layered 3 × 3 array with an aluminum frame.

To validate the design and feasibility of the two-layered array, prototypes of the two-layered cymbal arrays were fabricated, and their acoustic characteristics were measured. The individual cymbal transducer elements for the array were fabricated by firmly bonding the PZT-5A ceramic disk, PEEK ring, and fine-cut brass metal caps together with epoxy (EB-106, EpoxySet, Inc., Woonsocket, RI, USA) using a metallic fixture as described in [21]. Electrical wires were connected to both sides of the metal cap. Fabricated cymbal specimens were coated with a rubber layer for water-proof insulation. To fabricate the two-layered array as shown in Figure 2b), an aluminum frame was designed to support the cymbal transducers in their positions. The thickness of the frame was 2 mm, which corresponds to the vertical spacing of 0.04*λ* from Section 2.1. The cymbal elements were adhered to the frame using epoxy. The finite element model of the array with the frame is shown in Figure 16.

**Figure 17:**
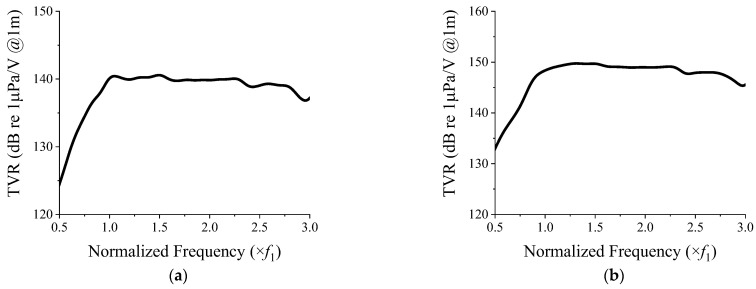
TVR spectra of the two-layered cymbal arrays with a frame: (**a**) 3 × 3 array; (**b**) 5 × 5 array.

The 3 × 3 array was used to illustrate the wideband spectrum; however, the design is applicable to higher-order arrays. Hence, the TVR spectra of the 3 × 3 and 5 × 5 two-layered arrays with frames were analyzed using the FEM, and are presented in Figure 17.

**Figure 18:**
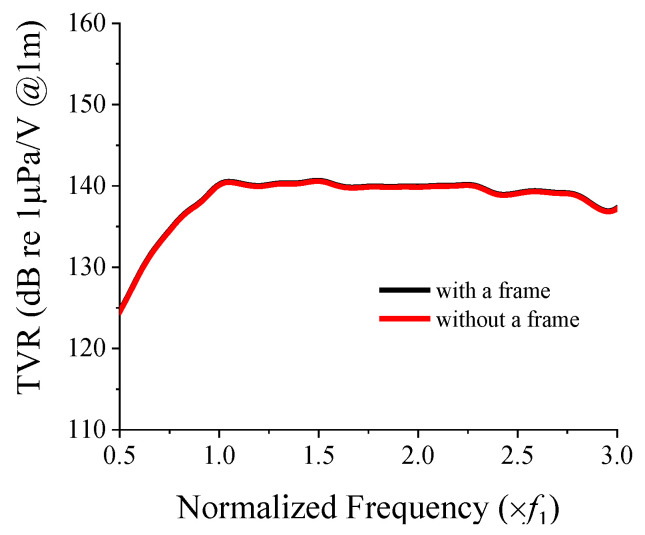
TVR spectrum of the two-layered 3 × 3 cymbal array with and without a frame.

Figure 18 presents the comparison of the results shown in Figure 17a and Figure 5b for the two-layered 3 × 3 array with and without a frame, respectively, for a CTC spacing of 0.2*λ*. From the figure, we can conclude that the effect of the frame on the TVR spectrum of the array is negligible.

**Figure 19:**
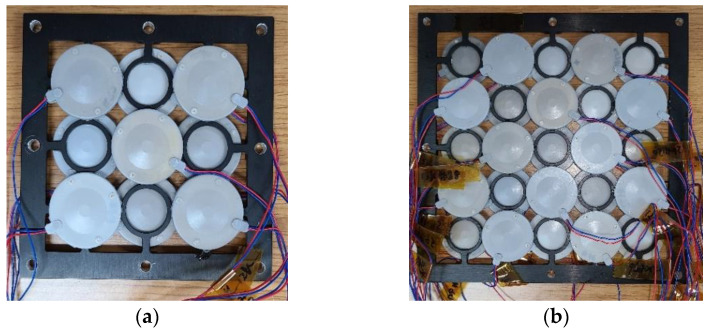
Prototypes of the two-layered cymbal arrays: (**a**) 3 × 3 array; (**b**) 5 × 5 array.

The fabricated prototypes of the two-layered 3 × 3 and 5 × 5 arrays with frames are shown in Figure 19.

**Figure 20:**
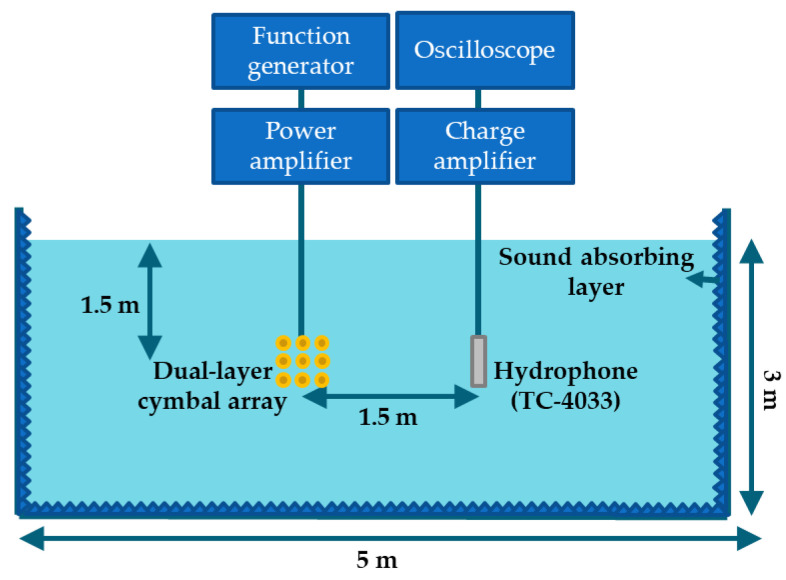
Schematic diagram of the experimental setup for TVR measurement.

The experiment was conducted in a free-field environment in a water tank 5 m in length and 3 m in depth. All of the inner surfaces of the water tank were covered with sound absorbing layers to prevent reflection of the waves transmitted by the array. The TVR of the array was measured at a depth of 1.5 m in the water tank by placing a hydrophone (Hydrophone TC4033, Teledyne RESON, Slangerup, Denmark) 1.5 m away from the array as shown in Figure 20. An electric impulse signal was applied to the array, and the charge from the hydrophone due to the sound pressure wave radiated from the array was amplified and used to calculate the transmitting voltage response (TVR) spectrum of the cymbal array [8,20]. During the measurement, time gating was used to catch only the first-arriving wave from the array at the hydrophone, excluding any later-arriving waves, such as those reflected by walls of the water tank. A quantitative comparison of the acoustic characteristics based on the measured and analyzed data is presented in Table 5.

As shown in Table 5, the measurement results matched well with the FEA results, which validated the wideband spectrum using the two-layered array. The difference between the measured and analyzed FBs for the 3 × 3 array was 3.4%, whereas it was less than 0.1% for the 5 × 5 array.

**Figure 21:**
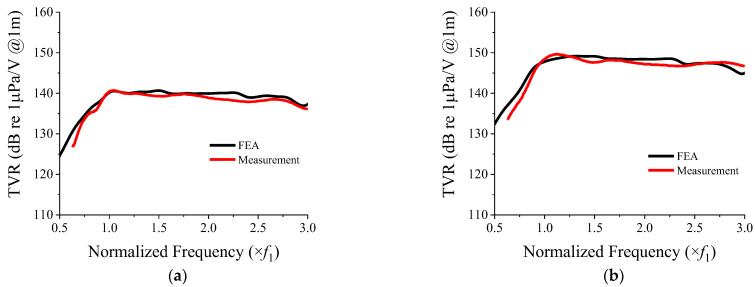
Comparison between measured and calculated TVR spectra of the two-layered cymbal arrays: (**a**) 3 × 3 array; (**b**) 5 × 5 array.

The results in Figure 21 proved that the analysis and simulation conducted in this study were accurate. The feasibility of the two-layered structure for achieving a wide bandwidth were verified. Improved interaction between elements in the array in the dual-layer structure resulted in a wider bandwidth, which could be used for accurate detection in sonar systems.

## 6. Conclusions

A dual-layer structure of a cymbal array was proposed. The effect of CTC spacing on the TVR spectrum based on the proposed array arrangement was analyzed. This effect was elucidated based on the mutual radiation impedance between the elements in the array. The CTC spacing between the elements was optimized for the maximum FB with a constraint on the peak TVR level. The FB of the optimized two-layered array was 34% greater than that of the one-layered array, thus justifying the advantage of the dual-layer array. The analysis was validated by comparing the results with the measurement data of two-layered 3 × 3 and 5 × 5 array prototypes. Using the two-layered arrangement, we achieved a wider bandwidth for the cymbal array while maintaining the TVR level over a required level.

The wideband cymbal arrays investigated in this work can be used for better underwater communication, both as projectors for transmitting data in a shorter time, and as wideband receivers. They are essential for modernized underwater wireless sensor network systems. The wideband dual-layer cymbal array structure developed in this study can be applied to other types of arrays as well, whose CTC spacing is inversely proportional to the bandwidth and is constrained by the size of the transducer. In future work, we will apply the dual-layer structure to the development of higher-dimension cymbal arrays for practical underwater communication applications.

## Figures and Tables

**Table 1:** Dimensions of structural parameters of the cymbal transducer.

Structural Parameter	Symbol	Dimension (mm)
Thickness of the piezoceramic disk	*t*_d_	1.0
Radius of the piezoceramic disk	*r*_d_	10.0
Apex radius of the metal cap	*r*_a_	2.3
Base radius of the metal cap	*r*_b_	8.2
Thickness of the metal cap	*t*_m_	0.5
Height of the cavity	*h*_c_	0.8
Width of the epoxy bond line	*w*_e_	0.3
Width of the ring	*w*_p_	3.0

**Table 2:** Acoustic characteristics of the 3 × 3 cymbal array vs. vertical spacing.

Vertical Spacing	Center Frequency (×*f*_1_)	−3 dB Bandwidth (×*f*_1_)	FB (%)
0	1.45	1.14	79.2
0.03*λ*	1.44	1.11	77.6
0.04*λ*	1.44	1.11	77.6
0.05*λ*	1.46	1.14	78.4
0.06*λ*	1.45	1.12	77.6
0.07*λ*	1.44	1.08	75.1
0.08*λ*	1.44	1.04	72.5

**Table 3:** Acoustic characteristics of the 3 × 3 two-layered cymbal array for various CTC spacings.

CTC Spacing	Center Frequency (×*f*_1_)	Maximum TVR (dB)	FB (%)
0.50*λ*	1.03	149.1	19.4
0.48*λ*	1.02	148.4	20.4
0.44*λ*	1.03	148.0	21.8
0.40*λ*	1.06	147.9	23.9
0.36*λ*	1.06	146.8	27.2
0.32*λ*	1.09	145.8	32.0
0.28*λ*	1.15	144.4	41.4
0.24*λ*	1.35	142.6	66.4
0.22*λ*	1.63	141.6	90.2
0.20*λ*	1.88	140.7	105.9
0.18*λ*	1.88	139.9	105.0
0.16*λ*	1.85	139.8	97.3

**Table 4:** Comparison of acoustic characteristics of one- and two-layered cymbal arrays.

	Center Frequency (×*f*_1_)	Maximum TVR (dB)	FB (%)
3 × 3 one-layered array (with closest CTC spacing—0.23*λ*)	1.45	141.9	79.2
3 × 3 two-layered array (with optimized CTC spacing—0.2*λ*)	1.88	140.7	105.9

**Table 5:** Quantitative comparison of acoustic characteristics of the two-layered 3 × 3 and 5 × 5 cymbal arrays based on FEA and measurement.

	Center Frequency (×*f*_1_)	Maximum TVR (dB)	FB (%)
3 × 3 (FEA)	1.88	140.7	105.9
3 × 3 (measurement)	1.88	140.7	102.4
5 × 5 (FEA)	1.87	149.8	105.1
5 × 5 (measurement)	1.96	149.7	105.2

## Data Availability

Not applicable.
